# Avian Reovirus: From Molecular Biology to Pathogenesis and Control

**DOI:** 10.3390/v16121966

**Published:** 2024-12-23

**Authors:** Islam Nour, Sujit K. Mohanty

**Affiliations:** Southeast Poultry Research Laboratory, U.S. National Poultry Research Center, United States Department of Agriculture-Agricultural Research Service (USDA-ARS), Athens, GA 30605, USA; islam.mohamed@usda.gov

**Keywords:** avian reovirus, replication, pathogenesis, epidemiology, immune response, vaccine

## Abstract

Avian reoviruses (ARVs) represent a significant economic burden on the poultry industry due to their widespread prevalence and potential pathogenicity. These viruses, capable of infecting a diverse range of avian species, can lead to a variety of clinical manifestations, most notably tenosynovitis/arthritis. While many ARV strains are asymptomatic, pathogenic variants can cause severe inflammation and tissue damage in organs such as the tendons, heart, and liver. In broilers and turkeys, ARVs can induce severe arthritis/tenosynovitis, characterized by swollen hock joints and lesions in the gastrocnemius tendons. Additionally, ARVs have been implicated in other diseases, although their precise role in these conditions remains to be fully elucidated. In recent years, ARV cases have surged in the United States, emphasizing the need for effective control measures. Routine vaccination with commercial or autogenous vaccines is currently the primary strategy for mitigating ARV’s impact. Future research efforts should focus on enhancing our understanding of ARV-induced pathogenesis, identifying host factors that influence disease severity, and developing novel vaccines based on ongoing surveillance of circulating ARV strains. This review aims to explore the molecular aspects of ARV, including virus structure, replication, molecular epidemiology, the roles of its encoded proteins in host pathogenesis, and the immune response to ARV infection. Furthermore, we discuss the diagnostic approaches of avian reovirus and the potential biosecurity measures and vaccination trials in combating ARV and developing effective antiviral strategies.

## 1. Introduction

**Figure 1:**
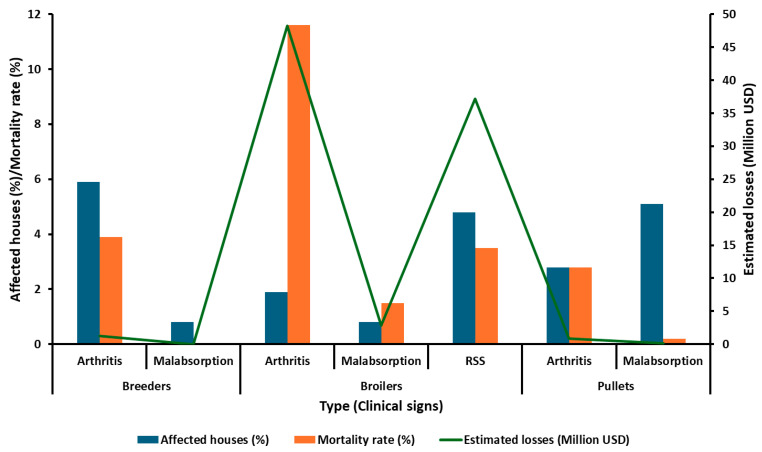
Estimated impact of reovirus on breeders, broilers, and pullets in the US for 2022.

Avian Reovirus (ARV) poses a significant threat to the poultry industry, infecting both domestic and wild avian species. ARV infections manifest in a diverse range of clinical signs, encompassing tenosynovitis/arthritis, immunosuppression, enteric disease, hepatitis, myocarditis, malabsorption, and runting–stunting syndrome [1,2]. While domestic avian species are generally susceptible to ARV, meat-type chickens and turkeys [3,4] are, however, more susceptible to viral arthritis/tenosynovitis caused by ARV than other species such as broiler/layer breeders [5,6], ducks [7,8,9], geese [7,10], and quail [11,12,13]. Often, the subclinical nature of ARV infections can lead to substantial economic losses due to diminished productive parameters, such as reduced weight gain, impaired flock uniformity, decreased feed conversion rates, increased lameness-related condemnations in processing plants, and compromised animal welfare [14,15]. As illustrated in Figure 1, the impact of ARV on the poultry industry is substantial.

The reovirus outbreak in California in 2015 led to alarmingly high morbidity and mortality rates [16]. Despite decades of routine vaccination with traditional strains (S1133, 1733, and 2408) implemented since the 1970s, the emergence of vaccine-resistant ARV isolates is suspected as a primary factor driving this outbreak. The inherent high mutation rate and recombination potential of RNA viruses [16], combined with the development of immune escape variants under selective pressure from these vaccines, has contributed to the decline in vaccine efficacy. Consequently, a comprehensive characterization of circulating ARV strains is crucial for developing effective control strategies. Furthermore, the increased use of autogenous vaccines has been associated with positive selection, leading to a diverse range of virulent strains. For instance, arthritis/tenosynovitis-causing ARV strains belonging to genetic cluster (GC) VI were recently reported in North Carolina [17]. The emergence of these strains may be linked to the use of autogenous vaccines containing a single GC V variant and two GC I isolates in breeder flocks. This practice may have inadvertently favored the fitness of GC VI strains over others [17].

ARV has a very high global prevalence, with most studies indicating that it is present in nearly all poultry flocks worldwide, meaning the prevalence rate is essentially considered “ubiquitous” or close to 100% in commercial poultry populations [18,19]; studies often report positive detection rates in the vast majority of flocks sampled, with some studies showing positive results in over 90% of flocks [20]. ARV has emerged as a significant concern within the poultry industry in the US, particularly in turkey production. The 2022 American Association of Avian Pathologists (AAAP) Research Priorities survey identified ARV as a top-ten research priority for the broiler industry and the number-one priority for turkey production [21]. This prioritization is further underscored by the 2023 United States Animal Health Association (USAHA) report, which ranks ARV-related issues among the top ten challenges facing the industry [22]. Notably, turkey arthritis and hepatitis have experienced a substantial increase in prevalence, with turkey arthritis rising 187% in 2023 alone [22]. Turkey hepatitis, caused by a newly identified ARV strain, has emerged as a major concern since 2020, primarily affecting poults [23]. Emerging evidence suggests a link between early-life hepatitis and the development of turkey reoviral arthritis later in the production cycle [21]. The economic impact of ARV on the turkey industry is substantial, with a 2019 National Turkey Federation (NTF) survey estimating potential losses of up to $33.7 million due to highly pathogenic ARV strains [21,22,23].

## 2. History

ARV, a significant pathogen of poultry, was first described in Muscovy ducks in South Africa in 1950 [24]. Subsequent isolations were reported in France in 1972 [25] and in broilers in the United States in 1957 [26]. The latter was initially misdiagnosed as a poxvirus due to its double-stranded nucleic acid [27]; it was later identified as a reovirus by electron microscopy [28]. The Fahey-Crawley virus, isolated in 1954 from chickens with chronic respiratory disease [29], was found to induce lesions similar to those caused by the viral arthritis agent, later defined as avian reovirus [30,31]. In addition to tendon sheath lesions, a pannus formation in the synovial membrane resembling rheumatoid arthritis in humans was also observed. Experimental infections in chickens demonstrated the virus’s ability to cause arthritis, tenosynovitis, and enteric diseases [30,31,32]. ARV has also been implicated in various other poultry diseases. These include blue comb disease in turkeys [33,34,35], myocarditis, and hepatitis in chickens [36,37,38].

Multiple studies have implicated turkey reoviruses (TRV) as the etiological agent of poult enteritis, a disease affecting young turkeys [39,40,41,42,43]. Additionally, TRV has been isolated from the joints and ruptured tendons of turkeys exhibiting tenosynovitis/arthritis, suggesting a potential association with this condition [44,45]. Nevertheless, experimental challenges with TRV have sometimes failed to reproduce tenosynovitis/arthritis in turkey models [46].

In 1997, an outbreak of duck reovirus (DRV) disease occurred in China, resulting in liver damage [47]. Reovirus isolations from chickens with arthritis, tenosynovitis, or enteric lesions have been reported globally [2,15,48,49]. The enteric diseases associated with ARV infection were first reported in 1966 [32]. Several established reovirus isolates, such as WVU 2937, Reo 25, and UMI 203, have been characterized [33,50,51]. Additionally, the S1133 reovirus isolate (referring to its diagnostic accession number) was identified from chickens with tenosynovitis in the United States [52]. Currently, several ARV isolates causing arthritis/tenosynovitis were characterized in different states in the USA [16,17,48,53]. In 2011, an outbreak of duck reovirus (DRV) occurred in Pekin ducks in China, characterized by the presence of large necrotic foci in the spleens of the affected ducks [54].

Serotyping studies have revealed the existence of multiple reovirus serotypes [55,56,57]. While isolates from the United States were found to be serologically related, some variant serotypes were identified in Europe [10]. In 2003, antibodies against goose reovirus (GRV) were detected in affected geese [10].

## 3. Classification

The Reoviridae family encompasses a diverse group of non-enveloped viruses with segmented double-stranded RNA genomes [58]. This family is further classified into two subfamilies: Sedoreovirinae and Spinareovirinae [59]. The Sedoreovirinae subfamily comprises six genera: Cardoreovirus (infecting crabs), Mimoreovirus (an environmental marine virus), Orbivirus (arthropod-borne), Phytoreovirus (infecting plants), Rotavirus (infecting humans and animals), and Seadornavirus (arthropod-borne, infecting humans and animals) [60,61,62,63,64].

The Spinareovirinae subfamily, on the other hand, consists of eleven genera: Aquareovirus (infecting aquatic species), Coltivirus (causing Colorado tick fever in humans), Cypovirus (infecting insects), Dinovernavirus (host unknown), Figivirus (infecting plants), Idnoreovirus (infecting insects), Mycoreovirus (infecting fungi), Orthoreovirus (infecting vertebrates), Oryzavirus (infecting rice), Piscinereovirus (infecting fish), and Crabreovirus (infecting mud crabs) [60,65,66,67,68,69,70,71,72,73,74].

Within the Orthoreovirus genus, six species have been identified: mammalian orthoreovirus (MRV), avian orthoreovirus or avian reovirus (ARV), baboon orthoreovirus, Nelson Bay orthoreovirus, piscine orthoreovirus, and reptilian orthoreovirus.

## 4. Virus Structure

### 4.1. Virus Genome

**Figure 2:**
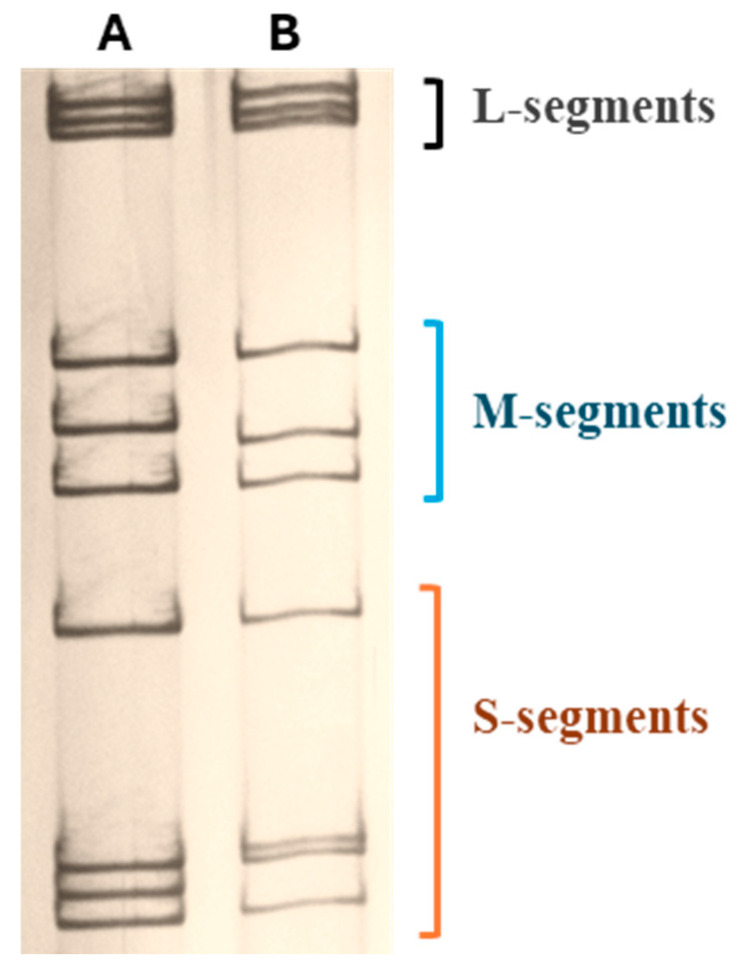
Electrophoretic mobility of genomic segments of avian reoviruses. (**A**) 2177 strain, (**B**) the vaccine strain S1133.

ARVs possess a segmented double-stranded RNA genome consisting of ten segments classified into three size classes: L-class (L1–L3; for large segments), M-class (M1–M3; for medium-sized segments), and S-class (S1–S4; for small segments) [75]. While segment S1 exhibits electrophoretic mobility closer to M3 (Figure 2), it is traditionally designated S1 to align with mammalian reovirus nomenclature, emphasizing the shared genomic organization of three M and four S genes, including the S1-encoded σ protein responsible for cell attachment.

The nucleotide sequences of several ARV strains’ genomic segments have been elucidated. Notably, with the exception of S1, each segment encodes a single primary translation product. The positive strand of each segment, identical to its corresponding mRNA, is capped at the 5′ end, while the negative strand possesses a 5′ pyrophosphate group [76]. A conserved 5′-terminal heptamer (GCUUUUU) and 3′-terminal pentamer (UCAUC) are present in all sequenced avian reovirus-positive strands, potentially serving as regulatory signals for transcription, replication, and/or encapsidation. Similar to other Reoviridae members, co-infection of cells with two avian reovirus isolates results in reassortment, generating progeny viruses containing genomic segments from both parental strains. This phenomenon is essential for dissecting the phenotypic contributions of individual genome segments.

### 4.2. Virus Proteins

ARVs express a complex proteome. Twelve primary translation products have been identified, eight of which are structural proteins incorporated into virions, while the remaining four are nonstructural proteins expressed in infected cells but absent from mature virions [77,78]. The L-, M-, and S-class genes encode proteins designated lambda (λ), mu (μ), and sigma (σ), respectively. The primary structural proteins include eight proteins, designated λA, λB, λC, μA, μB, σA, σB, and σC, to distinguish them from their mammalian counterparts (λ1, λ3, λ2, etc.). Notably, the M-class protein μB undergoes post-translational cleavage to yield μBN and μBC [79]. In addition to structural proteins, ARVs produce several nonstructural proteins. The M3 and S4 genes encode μNS and σNS, respectively [77,80]. Recent findings have identified another nonstructural protein, μNSC, which arises from the cleavage of μNS [81]. Furthermore, the tricistronic S1 gene encodes two additional nonstructural proteins, p10 and p17, as well as the structural protein σC [82,83].

The correspondence between gene and protein sizes is generally close, with the exception of the S1-encoded genes [77]. This multicistronic arrangement, with three overlapping open reading frames, allows for the expression of multiple proteins from a single transcript [84].

#### 4.2.1. Lambda-Class Proteins

**Figure 3:**
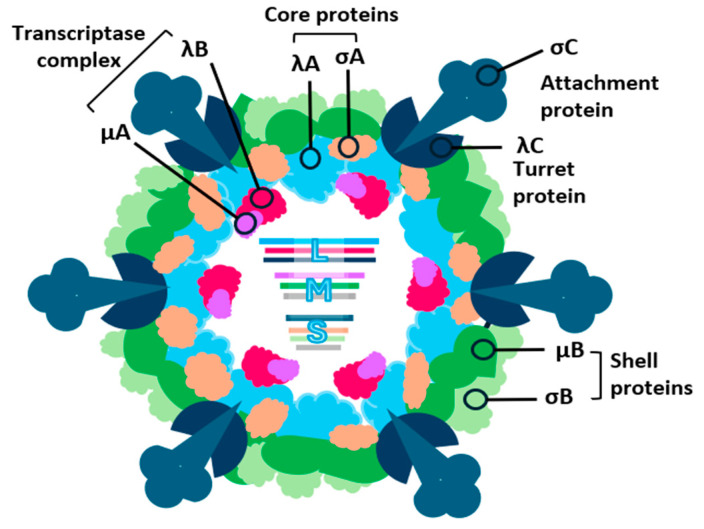
Diagrammatic structure of avian reovirus.

The protein λA (L1 gene) forms the inner core–shell, providing a scaffold for subsequent core assembly (Figure 3). While diffusely distributed in the cytoplasm when expressed alone, λA becomes associated with viral factories when co-expressed with μNS, suggesting that μNS mediates its recruitment [85]. The amino-terminal region of λA, a distinct hydrophilic domain, likely adopts an extended arm-like conformation in a manner similar to the mammalian λ1 [86].

The λB (L2 gene) is a minor core protein that functions as the viral RNA polymerase. This is supported by sequencing data of the L2 gene, which reveals the presence of RNA polymerase-specific motifs within the amino acid sequence of λB, confirming its role in viral replication. The λB polymerase motifs required for the concise template nucleosides positioning (at amino acids 515–529 and 583–588), NTP priming (at 557–568), and RNA polymerase activity (at aa 728–735) were highly conserved in chicken reovirus [17] and very similar to those in turkey reoviruses [87].

On the other hand, λC (L3 gene) spans the inner core and outer capsid. It assembles into pentamers, forming the turrets projecting from the five-fold axes of the core [78,88]. λC has been identified as the viral capping enzyme [76]. The amino-terminal 42 kDa fragment of λC possesses autoguanylylation activity with 169/188 K residues essential for guanylyl transferase activity, while the C-terminal 100 kDa fragment is dispensable for this function [89]. The methyltransferase S-adenosyl-L-methionine (SAM)-binding pocket in the λC protein was previously detected at aa residues 822–830 [87]. Comparative analysis of λC with mammalian and grass carp reovirus capping enzymes shows a high degree of amino acid sequence conservativeness, suggesting that it likely possesses both guanylyltransferase and methyltransferase activities, which are essential for mRNA capping [89].

#### 4.2.2. Mu-Class Proteins

The minor inner capsid protein μA, encoded by the M1 gene [78], remains largely uncharacterized. Structural predictions for μA suggest it can be divided into four distinct regions: an N-terminal domain (residues 1–149), a variable region (residues 150–462), an α-helix-rich domain (residues 463–615), and a C-terminal domain (residues 616–732) [90]. These predicted domains bear similarity to those of the MRV μ2 protein [91]. Given the hypothesized role of the μ2 protein being an RNA-dependent RNA polymerase cofactor, it has been proposed that μA may function as a transcriptase cofactor, interacting with the RNA polymerase λB to form the active transcriptase complex. Recently, μA was also reported to contain the 458-LALDPPF-464 motif that is similar to the N-6 adenine-specific DNA methylase [17,92,93].

**Figure 4:**
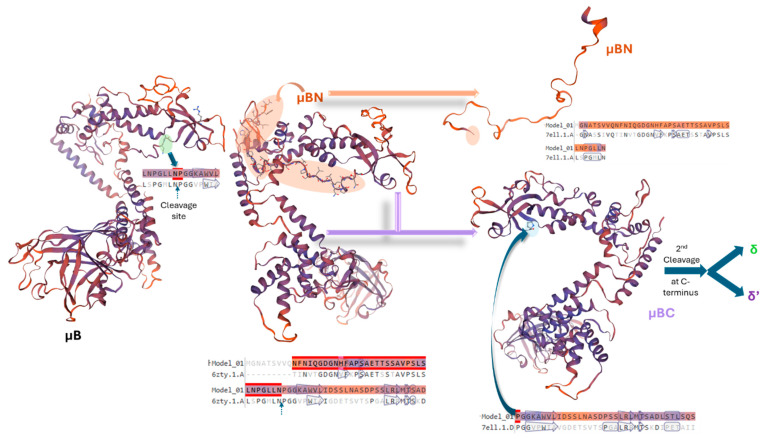
Posttranslational modification of the avian reovirus major capsid protein μB.

The M2 gene product, μB, is an N-myristoylated protein that undergoes cleavage near its N-terminus to generate μBN and μBC [77,78,79]. Both μB and its cleavage products are structural components of the outer capsid. Cleavage of μB occurs within a sequence (Asn-42 and Pro-43) resembling cleavage sites in mammalian reovirus μ1 and poliovirus polyprotein VP0, suggesting a conserved mechanism (Figure 4) [79]. However, this cleavage appears to require a viral factor, as it does not occur in the absence of other viral proteins. A likely candidate for the protease is σB, which forms a complex with μB and μBC [85]. In addition to its structural role, μBC is involved in viral entry and uncoating. Sequential cleavages of μBC generate δ and δ’, which are thought to facilitate interactions with lysosomal membranes and conformational changes necessary for core particle release [92,94]. Overall, μB is required for virus stability, membrane association affinity, and binding capacity, thereby affecting the reovirus replication and infectivity [79,95].

The M3 gene encodes the nonstructural protein μNS, which contains two predicted coiled-coil regions suggesting potential oligomerization [81]. μNS is cleaved near its N-terminus to produce μNSN (15 kDa) and μNSC (55 kDa), although the protease and the functional significance of this cleavage remain to be elucidated [86]. μNS is unique among viral proteins in its ability to form inclusions when expressed alone, indicating its crucial role in viral factory formation and early morphogenesis [81]. μNS interacts with σNS and λA, but not other viral proteins, suggesting non-competitive specific binding sites within μNS [85].

#### 4.2.3. Sigma-Class Proteins

The σA protein (S2 gene), a key component of the core–shell, exhibits high-affinity, sequence-independent binding to double-stranded RNA (dsRNA). This interaction is robust, as evidenced by the resistance of the σA-dsRNA complex to dissociate under high-salt conditions [96,97,98]. Disruption of the dsRNA binding site impairs σA’s ability to enter the nucleus, consequently inhibiting viral replication [99,100]. Notably, the arginine residues at positions 155 and 273 are critical for this process, as the σA R155/273A mutant fails to localize to the nucleolus, resulting in reduced ATP production and impaired viral replication [101]. Beyond its role in viral replication, σA has been implicated in viral evasion of the host interferon response. By sequestering dsRNA, σA inhibits the activation of protein kinase R (PKR) [96,102]. Furthermore, σA plays a crucial role in viral morphogenesis, stabilizing the core–shell λA protein and promoting the assembly of the outer capsid [103].

The S3-encoded protein σB is a major constituent of the outer capsid [76]. Unlike its mammalian counterpart σ3, σB does not bind dsRNA, suggesting a lack of anti-interferon activity [98]. This protein forms a stable tertiary complex with μB and μBC, which is essential for efficient virion assembly [81,85]. While σB is a critical component of the outer capsid, its specific functions within the virion remain to be fully elucidated. The σB protein, an outer capsid protein, includes neutralizing epitopes that are group-specific [104].

The minor outer capsid protein σC, encoded by the 3′-proximal cistron of the S1 gene, serves as the viral cell attachment protein. It binds to host cell receptors, triggering viral entry. Interestingly, σC has also been implicated in inducing apoptosis in transfected cells, although its role in virus-induced cell death remains unclear. The trimeric form of σC is necessary for its cell attachment activity. The σC protein, a key viral antigen, elicits the production of neutralizing antibodies during infection. Notably, this protein exhibits significant amino acid sequence variability among closely related strains [105,106,107,108]. This potential divergence has compromised the efficacy of conventional ARV vaccines in controlling viral arthritis within the poultry industry. To mitigate this challenge, molecular diagnostics are essential for identifying circulating ARV strains and guiding informed vaccination strategies [109].

**Figure 5:**
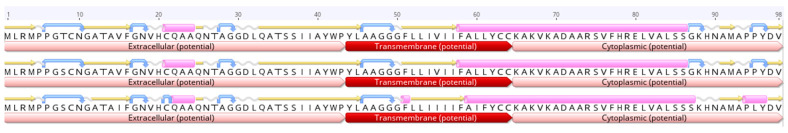
Two-dimensional secondary structure of avian reoviruses P10 fusogenic protein showing the three domains: the ectodomain, with potential extracellular exposure, and the endodomain, within the cytosol, intervened by the transmembrane domain. The color coding of the secondary structures is as follows: yellow arrows are β-strand, blue curved arrows are turns, white spirals are coil structure, and pink barrels are α-helix.

The S1-encoded p10 protein (10.3 kDa) is a type I transmembrane glycoprotein characterized by a central hydrophobic domain that anchors it to the cell membrane [110]. The ectodomain, exposed to the extracellular milieu, and the endodomain, situated within the cytoplasm, are separated by this transmembrane domain (Figure 5). Upon expression, p10 triggers cell–cell fusion, a process facilitated by a specific di-cysteine motif (63/64C) at the end of the transmembrane domain and before the cytoplasmic domain [111]. In addition, p10 exhibits membrane-destabilizing properties, which may play a crucial role in viral entry and dissemination [112]. The N-terminal region of p10, the fusogenic extracellular domain, is indispensable for its membrane-permeabilizing activity. Deletion of this domain completely abolishes its fusogenic capacity while leaving its ability to associate with cellular membranes unaffected [112].

The S1-encoded p17 protein is a nucleocytoplasmic shuttling protein that localizes to the nucleus via a nuclear localization signal [113]. It exhibits DNA-binding activity, suggesting a potential role in viral gene expression or host cell regulation. Recent studies have linked p17 to cell cycle arrest and the activation of p53 and p21 as well as the inhibition of the CDK2/cyclin A2 complex, leading to Akt S473 phosphorylation inhibition, indicating a possible role in modulating host cell responses to infection [114,115].

The nonstructural protein σNS, encoded by the S4 genome segment, is a non-sequence-specific single-stranded RNA (ssRNA)-binding protein [98,116,117]. Within infected cells, it associates with viral RNA to form ribonucleoprotein complexes, implicating its involvement in RNA metabolism. While σNS can form both homodimers and homotrimers, it is primarily localized to viral replication factories [85,86]. Although its exact functions remain elusive, σNS is likely to play a crucial role in viral RNA packaging and replication. The ssRNA-binding activity of σNS is essential for its function, and any alterations to this activity can significantly impact viral packaging, replication efficiency, and ultimately, the pathogenicity of the virus [118]. The N-terminal region of σNS, encompassing the first 38 amino acid residues, has been identified as a critical determinant of RNA binding [119]. Specifically, two positively charged residues, arginine 6 (R6) and arginine 29 (R29), within this N-terminal region are indispensable for the protein’s RNA-binding capacity [120].

### 4.3. Virus Particle

ARVs are nonenveloped, icosahedral particles with a diameter of 85 nm and a buoyant density of 1.37 g/mL, containing ten double-stranded RNA segments [75,88]. Biochemical analyses have revealed the distribution of structural proteins within the two capsid shells of ARV strain S1133 [78,80]. The outer capsid comprises μB, μBC, μBN, σB, and σC, while the core contains λA, λB, μA, and σA. Protein λC is unique in that it extends from the inner core to the outer capsid, forming the 12 pentameric core turrets through which newly transcribed viral mRNAs exit the particle and acquire their 5′ cap structures [78,80].

Electron cryomicroscopy, three-dimensional image reconstruction, and X-ray diffraction studies have provided detailed insights into the structure of mammalian reovirus particles, revealing the arrangement, interactions, and functions of viral structural proteins [121,122,123,124,125,126,127]. In contrast, the structure of avian reovirus particles has been less extensively studied. A previous study by Zhang et al. (2005) examined the structure of avian reovirus 138 using electron cryomicroscopy and image reconstruction, revealing strong structural similarities to mammalian reoviruses [88]. The ARV particle has a central core containing the λA scaffold protein and concentric rings of dsRNA genome segments [121]. The core also houses transcriptase complexes composed of λB and μA, and 150 σA core nodules stabilize the λA shell. Twelve pentameric λC turrets project from the λA shell, forming cavities that serve as sites for mRNA capping. The outer capsid comprises μBC trimers and σB monomers, which form the base and knobby projections of the outer shell, respectively. The major structural difference between avian and mammalian reoviruses lies in the absence of “hub-and-spokes” structures and C-terminal sequences in the avian outer capsid proteins [86].

Intact ARVs can be purified from infected cells by a series of centrifugation steps, including freon extraction and equilibrium ultracentrifugation in cesium chloride gradients, yielding the viral band at 1.38 g.ml^−1^ density [128]. In addition to virions, empty viral particles lacking dsRNA genome segments were also observed close to the uppermost part of the tube. ARVs are unstable and sensitive to various environmental factors, including temperature and pH. They can be converted into intermediate subviral particles (ISVPs) or core particles through specific treatments, which can affect their infectivity and transcriptional activity [78,128]. Virus treatments are performed at temperatures of 37 °C with neutral pH and low concentrations of either trypsin or chymotrypsin (in the case of reovirus suspension in a 0.14 M NaCl solution) or above 40 °C using a hypotonic buffer. ISVPs, which lack outer capsid proteins but retain core proteins and λC and σC, are infectious but tend to aggregate. Core particles, which lack outer capsid proteins and σC, are transcriptionally active but non-infectious.

## 5. Strain Variation

Historically, ARVs have been classified based on serotypes or their relative pathogenicity in chickens [129]. Inoculating specific pathogen-free (SPF) chickens with antigenically similar viruses via various routes has revealed strain-specific differences in pathogenicity and viral persistence [1]. Based on clinical signs, mortality rates, weight loss, tissue lesions, invasiveness, and viral persistence, isolates have been categorized as low, intermediate, or high pathogenicity. Highly pathogenic isolates exhibit prolonged persistence in infected tissues and induce a rapid, sustained antibody response. All isolates, however, induce similar mortality rates in chicken embryos.

In recent years, a new class of antigenically and genetically distinct reoviruses has emerged from chicken and turkey flocks [17,48,109,130,131,132,133,134]. The pathogenicity of Chinese ARVs has evolved, complicating disease control measures [15]. A severe reovirus outbreak in California in 2015 resulted in exceptionally high morbidity and mortality [135]. Despite decades of routine vaccination with traditional strains (S1133, 1733, and 2408) dating back to the 1970s, the emergence of vaccine-resistant ARV isolates is suspected as a primary driver of this outbreak. The high mutation rate and recombination potential characteristic of RNA viruses [16], coupled with the development of immune escape variants under vaccine-induced selective pressure, have contributed to a decline in vaccine efficacy.

The pathogenesis of four enteric turkey reoviruses (TERVs; NC/SEP-R44/03, NC/98, TX/98, and NC/85) and one ARV (strain 1733) was investigated by infecting SPF poults [136]. The TERV isolates were derived from turkey flocks experiencing poult enteritis and were genetically distinct from previously reported ARVs. Although viral antigen was detected in the bursa of Fabricius and the intestine of poults inoculated with the virulent chicken-origin strain, no tissue lesions were observed. The TERVs exhibited similar tissue tropism but varied significantly in lesion severity. Poults infected with NC/SEP-R44/03 or NC/98 displayed moderate to severe bursal atrophy, while those infected with TX/98 or NC/85 presented mild to moderate bursal lymphoid depletion. Another study compared the pathogenicity of three turkey arthritis reoviruses (TARVs: TARV-MN2, TARV-MN4, and TARV-O’Neil) and one TERV. The O’Neil strain of TARV was the most pathogenic, inducing tenosynovitis and clinical lameness, followed by TARV-MN2 and TARV-MN4. The TERV-MN1 did not cause tenosynovitis [133].

## 6. Avian Reovirus Replication

### 6.1. Penetration and Uncoating

**Figure 6:**
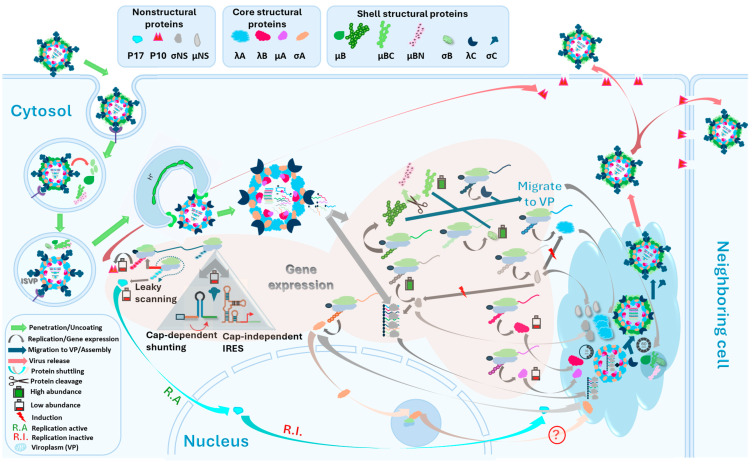
A diagrammatic representation of the avian reovirus replication cycle. Virus morphogenesis and release.

Avian reoviruses initiate infection by binding to specific cell surface receptors through interactions between the outer capsid protein σC and host cell receptors [84,128], as shown in Figure 6. While the precise nature of these receptors remains elusive, they were suggested to be proteinaceous and distinct from sialic acid, a receptor utilized by mammalian reoviruses. Interestingly, avian reoviruses exhibit a broader host range than their mammalian counterparts, infecting both avian and mammalian cells and implying a ubiquitous distribution of their receptor [137]. Conversely, the inability of mammalian reoviruses to infect avian cells underscores the specificity of receptor-virus interactions [138]. Saturation binding assays have revealed approximately 18,000 receptor sites per chicken embryo fibroblast cell [128].

Viral entry into the host cell necessitates membrane penetration. ARVs, being nonenveloped viruses, enter host cells via receptor-mediated endocytosis. Since σB forms a stable complex with μB and μBC during virion maturation [81,85], upon internalization, outer shell proteins are thought to be removed, promoted by the chicken’s body temperature (39.5 °C). It was observed that avian reovirus tends to lose their outer shell polypeptides when incubated at 40 °C [128]. Thereafter, the acidic endosomal environment triggers the proteolytic cleavage of the μB major outer capsid protein into δ and δ’ polypeptides and uncoating [94]. This uncoating process is essential for viral core release into the cytoplasm. In contrast, mammalian reovirus uncoating involves a single cleavage event, generating a δ polypeptide but not a δ’ polypeptide [139,140]. The specific role of the δ’ polypeptide in avian reovirus infection remains to be elucidated.

Endosomal acidification is a critical factor in avian reovirus uncoating and replication. Inhibitors of vacuolar H^+^-ATPase, such as bafilomycin A1 and concanamycin A, as well as lysosomotropic agents like ammonium chloride and chloroquine, block viral uncoating and replication when added at the onset of infection but not at later time points [141,142]. Additionally, inhibition of cysteine proteases, including cathepsins B, H, and L, using E64 also prevents viral uncoating and replication [142], highlighting the importance of lysosomal proteases in this process. Virus uncoating is then followed by the release of viral cores into the cytosol to initiate viral gene expression.

### 6.2. Viral Gene Expression

ARV gene expression initiates with the synthesis of all ten viral mRNAs by a virus-encoded dsRNA-dependent RNA polymerase. This polymerase, a core component, utilizes the negative-sense viral genome segments as templates for mRNA transcription [76]. While intact virions possess RNA polymerase activity, they cannot synthesize full-length transcripts in vitro, suggesting that the polymerase’s activity is regulated by the viral particle’s structural constraints. The minor core protein λB is believed to harbor the active site of the polymerase.

ARV exhibits a unique host range, and its replication in mammalian cells is restricted. Spandidos and Graham reported that only four of the ten viral genome segments of ARV S1133 are expressed in mouse L cells, leading to incomplete replication [143]. This observation, coupled with the temporal regulation of mammalian reovirus gene expression, suggests a transcriptional basis for this host restriction. Nanoyama et al. (1974) proposed a model involving a cellular factor that blocks transcription of “late” genes, which is subsequently inactivated by viral proteins encoded by “early” transcripts [144]. However, subsequent studies by Wiebe and Joklik [145] and Zweerink and Joklik [146] challenged this model, suggesting that the observed temporal expression pattern might be due to differential protein abundance rather than transcriptional regulation. Benavente and Shatkin (1988) and Mallo et al. (1991) further solidified the notion of non-temporal regulation by demonstrating the synthesis of all ten avian reovirus transcripts in mammalian cells [147,148].

ARV mRNAs are capped at their 5′ ends but lack 3′ poly(A) tails and contain short untranslated regions at both ends. They are synthesized within the inner core and acquire their caps through the channels formed by the λC capping enzyme, as displayed in Figure 6 [88]. These mRNAs serve as templates for both protein synthesis and genome replication [149]. The σNS protein binds to viral mRNAs with a binding site size of 10–20 nucleotides [98].

Most ARV mRNAs are monocistronic, with translation initiated at the 5′-most AUG codon. However, the *s1* mRNA is polycistronic, encoding three proteins from overlapping open reading frames: the nonstructural proteins P10 and P17 and the outer capsid protein σC [82]. Translation of the first two cistrons likely occurs through leaky scanning [150], while the third cistron may be translated by cap-dependent shunting [151] or cap-independent internal ribosomal entry [152] owing to the inaccessibility of ribosomal linear scanning imposed by the third cistron upstream sequences (complex leader sequences).

ARV protein expression is regulated primarily at the translational level. While all viral mRNAs are produced in similar amounts, the abundance of individual proteins varies significantly. For instance, μBC, σB, and σNS were noted as the most abundant proteins, whereas the λB, μA, and the three S1-encoded proteins were the least abundant [86]. The mechanisms underlying this differential expression and viral-induced host protein synthesis shutoff remain to be fully elucidated. The synthesis of negative-strand genomic RNAs is less well understood. It is presumed that viral mRNAs are encapsidated and serve as templates for negative-strand synthesis, with the λB protein likely playing a catalytic role [149].

Another possibility is that σNS-mediated RNA polymerase activity moderates the full-length negative-strand synthesis, to be discussed later [153]. Both σA and P17 proteins exhibit nuclear localization signals (NLSs) and accumulate in the nucleoplasm of infected and transfected cells [113]. However, P17 is excluded from the nucleolus, unlike σA. The nucleolar targeting of σA is mediated by two basic arginine residues, R155 and R273. Mutations at these residues impair both nucleolar targeting and the dsRNA binding capacity of σA [113]. The ability of σA to bind dsRNA and target the nucleolus suggests that its interaction with duplex structures of rRNA may drive its removal from the nucleoplasm and accumulation in the nucleolus, a mechanism similar to that observed for other nucleolar RNA-binding proteins [128,154,155,156,157]. σA was detected in both the cytoplasm and nucleolus as early as 6 h post-infection [100].

The p17 protein exhibits nucleocytoplasmic shuttling activity [113]. This protein modulates various cellular signaling pathways and interacts with multiple cellular proteins, leading to autophagosome formation, translation shutoff, and cell cycle arrest, which are conducive to viral replication [115,158,159,160,161]. The nucleocytoplasmic distribution of p17 is dynamically regulated by transcriptional activity, with nuclear localization observed upon transcriptional activation and cytoplasmic redistribution following transcriptional inhibition [113]. A monopartite-type nuclear localization signal (NLS) located near the C-terminus of p17 (amino acid residues 104 to 146) has been identified as both essential and adequate for nuclear import [113].

**Figure 7:**
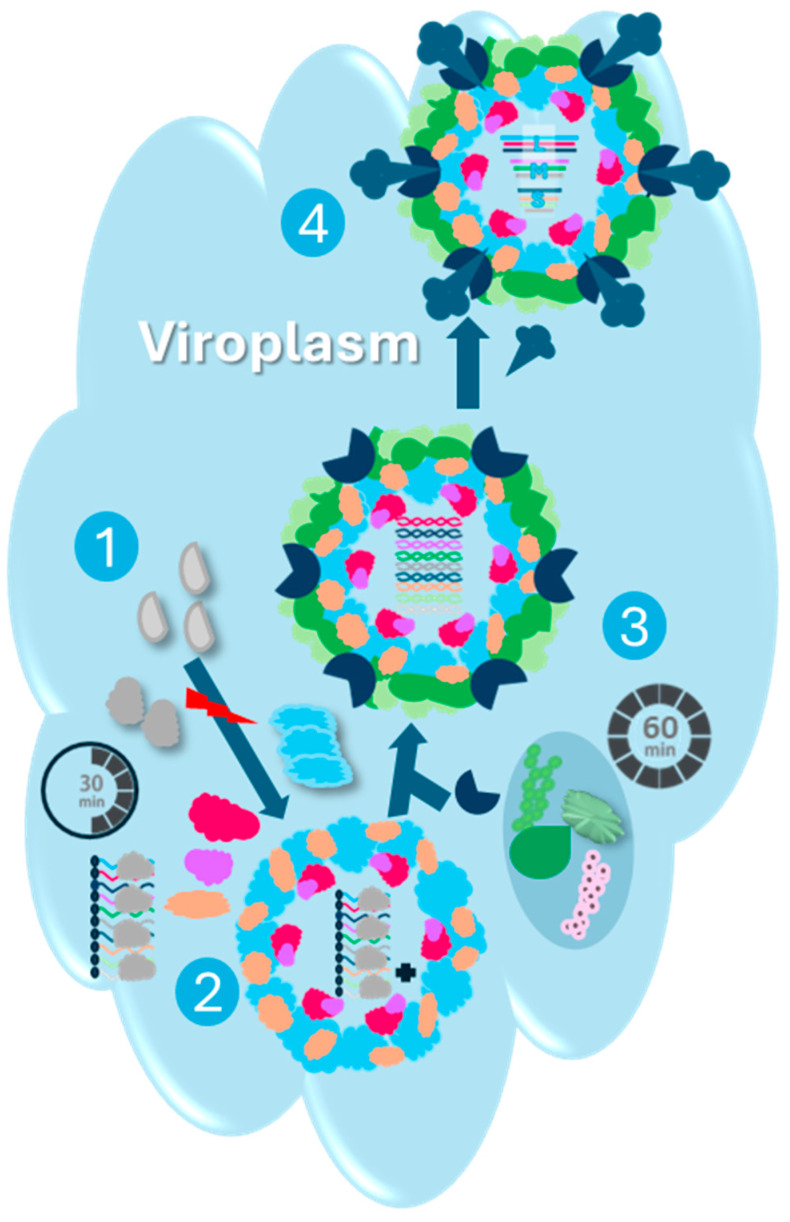
Avian reovirus assembly inside the viroplasm (cytoplasmic viral inclusions). Initially the M3-encoded protein (μNS) induces the viroplasm formation via the recruitment of the major core protein (λA) and the single-stranded RNA binding protein (σNS). Thereafter, the remaining core proteins are localized to the viroplasm, including λB, μA, and σA, within 30 min post-translation to form the core virus particle (step 2). Over 30 min later, the outer shell proteins are recruited to the viroplasm, with the primary assembly of the turret protein (λC), followed by the assembly of the minor capsid protein (σB) with the major capsid protein (μB) and its cleaved forms (μBC and μBN) forming a stable ternary heterocomplex prior to incorporation onto the core particle, as depicted in step 3, and eventually the introduction of viral attachment protein (σC) in step 4. Less is known about genome recruitment. However, the positive-stranded RNA strands of the virus genome are thought to be recruited prior to or during the encapsidation and are transcribed to produce the negative strands for the generation of the 10 genomic double-stranded RNA segments using the poly-C-dependent polymerase (σNS), as shown in step 2, followed by dissociation of σNS under high ionic strength once the dsRNA is generated in step 3. The color coding of the viral proteins is described in Figure 6.

The entire Reoviridae family assembles their virions within cytoplasmic phase-dense inclusions, often referred to as viral inclusions or viroplasms (Figure 7) [162,163,164]. These non-membrane-bound structures lack cellular organelles but harbor both structural and nonstructural viral proteins, as well as viral particles in various stages of assembly.

Electron microscopy of avian reovirus-infected cells has revealed the formation of large, perinuclear paracrystalline arrays of inclusions [103]. Immunofluorescence microscopy has further characterized these inclusions as globular structures that, unlike those of many mammalian reoviruses, are not associated with microtubules [81,85]. Expression studies have identified the μNS protein as the key determinant for inclusion formation in avian reovirus-infected cells. This protein selectively recruits σNS and λA, but not other viral proteins, to the inclusion sites [81,85]. While all viral proteins ultimately localize to inclusions, the precise timing and mechanisms of their recruitment remain to be fully elucidated.

To investigate the dynamics of viral protein incorporation into inclusions and virions, Tourís-Otero et al. employed a combined approach of metabolic pulse-chase radiolabeling, cell fractionation, and immunoprecipitation [85]. This study demonstrated that ARV morphogenesis occurs exclusively within the globular inclusions. The incorporation of viral proteins into both inclusions and virions is a selective and temporally regulated process. Core proteins are assembled within the first 30 min post-synthesis, followed by the addition of outer capsid proteins over the next 30 min to complete virion maturation. While the mechanisms underlying the specific packaging of viral mRNAs into progeny virions remain unclear, the RNA-binding activity of σNS and its early association with inclusions [81,98] suggest a potential role for this protein in this process. The σNS plays a role in the selective assortment of genomic segment precursors by enabling RNA–RNA interactions in viral inclusions consistent with the observed capacity of σNS to accelerate RNA folding, acting as an RNA chaperone [165,166,167]. A recent report demonstrated the capability of σNS to assemble in vitro into elongated hexamers, thereafter binding to ssRNA strands or segments and recruiting ssRNAs into viral inclusions with high nanomolar affinities, associated with the bound ssRNAs expansion, indicating its unwinding activity of the ssRNA secondary structure [165]. This σNS-derived RNA helix unwinding is similar to the helix destabilizing activity of rotavirus NSP2 [168] and gives rise to ribonucleoprotein complexes [165,169]. Unlike NSP2, which binds to ssRNAs irrespective of their secondary structure complexity, σNS exhibits less affinity toward potentially stable hairpins that could justify the selective segment assortment [170]. Moreover, it was reported that σNS has a poly(C)-dependent RNA polymerase activity, and therefore, it may generate complementary antisense strands [153]. The σNS-mediated helix unwinding endorses the complementary strands annealing with higher stability and prolonged complementarities of the resulting intermolecular duplexes [165]. Similar to RNA chaperones that dissociate upon the completion of the correct RNA folding, σNS detaches from the assorted RNAs before or during virus encapsidation in the viroplasm [171]. This could be owing to the low affinity of the σNS to the double-stranded RNA since the ssRNAs are annealed to their complementary strands [85,98,172].

On the other hand, the molecular mechanisms governing the release of ARV particles from infected cells are not fully understood. However, the nonstructural p10 protein, with its cell-permeabilizing and cell-fusion activities [110,112], is likely to play a significant role in both virus release and cell-to-cell spread. Additionally, the expression of the σC protein has been linked to the induction of apoptosis [173]. Furthermore, avian reovirus infection is characterized by the formation of cell–cell fusion (syncytia), a process primarily mediated by the P10 protein [174]. This phenomenon may also contribute to virus dissemination to neighboring cells.

## 7. Molecular Epidemiology and Transmission

ARVs have a broad host range, infecting various bird species. However, those associated with tenosynovitis and arthritis are primarily found in chickens and turkeys [175]. While ARVs have been isolated from clinically ill ducks, geese, pigeons, and psittacines, a significant host–pathogen relationship has only been established in geese and ducks [10,137]. These viruses exhibit remarkable environmental persistence, surviving in poultry house materials like wood, feathers, eggshells, and drinking water for extended periods [176]. This environmental resilience contributes to the persistence of ARV infections within poultry farms.

Factors influencing ARV transmission are multifaceted. Young birds are more susceptible to infection with clinical signs compared to older birds [177,178]. Transmission routes include vertical transmission through the egg [179,180,181] and horizontal transmission via the fecal–oral route [182]. Additionally, fecal contamination through broken skin may facilitate the entry of ARVs into leg tendons and joints [183]. Breed susceptibility varies, with heavy meat-type chickens being more commonly affected, though light egg layers and broilers can also experience infection and disease [184,185].

**Figure 8:**
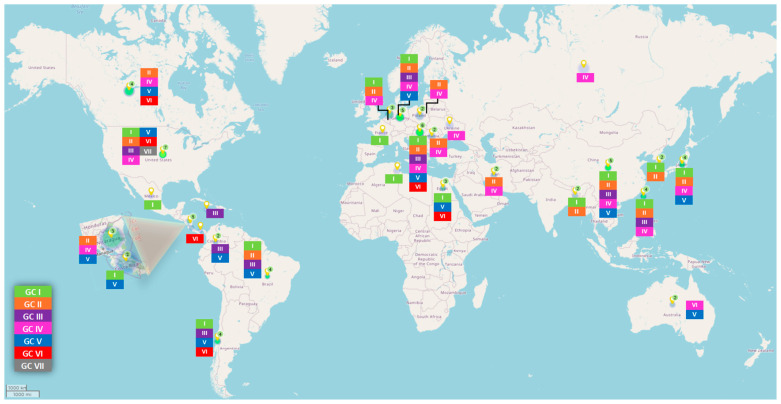
Global geographical distribution of genotypic clusters of avian reovirus. The seven σC-based genetic clusters (GCs) of ARV is referred as I–VII.

The ARV S1 segment encodes the σC protein, a minor capsid protein crucial for viral replication and pathogenesis [116,186,187], and has provided insights into viral diversity and evolution [107,188]. This protein facilitates early-stage infection by mediating virion-host cell interactions and inducing type-specific neutralizing antibodies [14,132,189]. Despite its significance, the association between σC and pathogenicity or antigenicity remains incompletely understood [16,190]. Nevertheless, σC has been widely used for ARV genotyping, with studies conducted in North America [20,191,192,193,194], Europe [106,134,195], and China [196,197] targeting the S1 gene. Six distinct genotypes based on σC have been identified globally [48,106,132,191]. Molecular characterization of ARV isolates from the USA has identified various genotypic clusters. Studies have reported the presence of GC I, II, III, IV, V, and VI in the USA (Figure 8) [16,17,48]. Phylogenetic analysis of ARV field strains from Pennsylvania poultry revealed six genotyping clusters [48]. Notably, one-third belonged to GC II, followed by about 24% and 22% of field strains clustered with GC V and GC I (containing the standard ARV vaccine strains, S1133, 1733, and 2048), respectively, with a minority belonging to clusters III, IV, and VI. In 2017 and 2018, a novel genotype cluster, GC VII, was identified, comprising seven distinct isolates [198]. These isolates shared less than 60% sequence identity with the previously characterized genotype clusters GC I–VI, indicating a significant genetic divergence. Notably, GC VII isolates have not been detected in subsequent samples submitted to the Public Health Diagnostic Reference Laboratory (PDRC). The prevalence of ARV-related diseases in South America has risen over the past decade, attributed to diverse pathogenic strains [132]. For instance, in Brazil, ARV infections have led to significant economic losses due to arthritis-related culling [192,199]. Initial studies identified GC II and V strains in Brazil. However, subsequent research in 2023 revealed additional genotypic clusters, including I and III, and subgenotypic clusters within I, II, and IV [200].

On the contrary, one or more of the five genotypic clusters (I–V) have been identified in various European countries, including France, Germany, the Netherlands, Spain, Hungary, Romania, and Ukraine [106,134,195,201,202]. GC I is the most prevalent cluster in Europe, encompassing diverse strains from various regions. Studies in specific European countries have revealed varying distributions of ARV genotypes. For example, in the Netherlands and Germany, GC I and IV were predominant, while in Hungary, GC II was the most common [106,134,195]. In Africa, ARV was initially identified in Egypt in 1984 [203] and has since been detected serologically in multiple governorates [204]. Both vaccinated and non-vaccinated flocks in Egypt exhibit high ARV prevalence. Notably, ARV strains isolated in Egypt belong to GC V, distinct from the GC I vaccination strains [205]. In Tunisia, ARV isolates were classified as GC I, sharing similarities with strains from China, England, Japan, and Canada [206].

Moreover, tenosynovitis/arthritis syndrome caused by ARV has been a significant concern in Asia, particularly in countries like China, Korea, and the Middle East [201,207]. In Taiwan, ARV isolates have been identified in GC I, II, III, and IV [208]. High ARV seroprevalence has been reported in broiler breeders in Turkey (95.83%) [209], Swiss poultry flocks (98.5%) [210], and Iranian poultry flocks (98.3%) [211]. In India, the overall ARV prevalence was 8.67% [212]. Genotypic analyses have revealed diverse ARV strains often differing significantly from vaccine strains. This genetic diversity underscores the challenges in developing effective control measures [16,213].

Tenosynovitis/arthritis emerged as the most prevalent clinical manifestation across all genotypic clusters, underscoring its ubiquitous nature (Table 1). Notably, GC I and IV demonstrated a wider global distribution, suggesting their significant impact on poultry populations. While GC I is generally associated with tenosynovitis/arthritis, two isolates exhibited respiratory signs of infection, emphasizing the intra-cluster variability and the need for a comprehensive approach to clinical diagnosis.

In contrast to the relatively focused clinical profile of GC I and VI, GC IV and V presented a diverse range of clinical signs, including tenosynovitis/arthritis, runting–stunting syndrome, malabsorption, and various other manifestations. This heterogeneity complicates the identification of specific clinical markers for GC IV and V, highlighting the challenges in their diagnosis and control.

Conversely, GC VI exhibited a more consistent clinical presentation, primarily characterized by tenosynovitis/arthritis. This distinct clinical profile sets GC VI apart from other genotypic clusters, suggesting a more homogenous impact on infected poultry.

## 8. Immunity and Avian Reovirus-Mediated Immune Evasion

### 8.1. Humoral Immune Response Against Avian Reovirus

ARVs elicit a robust humoral immune response, with the production of group- and serotype-specific neutralizing antibodies detectable within 7–10 days post-infection [215]. Maternally derived antibodies can provide significant protection to day-old chicks against clinical signs of reovirus infection [216,217]. However, the level of protection afforded by these antibodies is influenced by various factors, including serotype similarity, virus virulence, host age, and antibody titer [108,218].

The extent of viral replication plays a crucial role in shaping the innate immune response and subsequent clinical disease. Studies have shown that avian reoviruses with higher multiplication rates induce significantly elevated levels of pro- and anti-inflammatory cytokines, such as IL-6, IL-10, and IFN-γ, compared to those with lower replication rates [219]. Additionally, avian reovirus infection can activate specific cellular signaling pathways, including PI3-kinase, NF-κB, and Stat-3, which contribute to inflammation mediated by IL-6 [220].

Interestingly, the detection of anti-nuclear and anti-collagen antibodies in the serum of reovirus-infected chickens suggests a potential link between avian reovirus infection and autoimmune responses [221,222].

### 8.2. Cell-Mediated Immune Response to Avian Reovirus

Upon ARV infection, birds initiate an innate immune response involving natural killer cells, dendritic cells, macrophages, and heterophils. Cytokine profiling, particularly of IL-1, IL-6, and TNF-α, has been employed to assess macrophage activation during this phase [223].

Subsequently, the innate response triggers lymphocyte proliferation, leading to the generation of T-cell and B-cell clones [223]. CD8+ T cells have been identified as key players in viral clearance, demonstrating superior efficacy compared to CD4+ T cells in combating ARV [224]. Notably, the temporal dynamics of lymphocyte infiltration during ARV infection exhibit a distinct pattern: acute infection (2–6 days post-infection, or dpi) is characterized by a predominant CD8+ T-cell response, subacute infection (8–14 dpi) involves both CD4+ and CD8+ T cells as well as IgM+ B cells, and chronic infection (>14 dpi) is associated with CD4+ T-cell infiltration and limited B-cell activity. This immunological profile bears a striking resemblance to the lymphocytic response observed in human rheumatoid arthritis [225], further solidifying the potential of ARV as a model system for studying this autoimmune disease [57]. ARV possesses the capacity to suppress lymphocyte proliferation [226,227], which may underlie the clinical immunosuppression often observed in ARV-infected birds.

Interferon production has been documented both in vitro and in vivo in response to ARV infection. The attenuated S1133 strain induces interferon in chick embryo cell cultures and in the lungs of infected birds, while pathogenic strains elicit serum interferon (IFN) levels [135,228]. The viral σA protein is believed to play a crucial role in evading interferon-mediated antiviral effects. In mice, inactivated ARV can induce IFN-dependent isotype switching, leading to IgG2a antibody responses [229].

### 8.3. Mucosal Immune Response

Mucosal immunity, particularly IgA, plays a crucial role in protecting the respiratory and digestive tracts of birds from viral infections, including avian reovirus (ARV). Previous exposure to ARV, either through natural infection or vaccination, can induce a mucosal IgA response that provides a first line of defense against subsequent challenges [223]. Maternal IgG antibodies can also contribute to early mucosal protection in young birds.

The magnitude and quality of the mucosal IgA response to ARV can be influenced by various factors, including the age of the bird and the route of inoculation. Studies have shown that older birds (1–3 weeks of age) produce higher titers of mucosal IgA compared to younger birds (1-day-old chicks) [230]. Additionally, oral administration of ARV induces a more robust mucosal IgA response than subcutaneous inoculation. In contrast, systemic IgG responses, as measured in serum, are not significantly affected by age or route of inoculation.

Recent research has highlighted the immunogenic potential of specific ARV proteins. The σC protein, for instance, has been shown to enhance both systemic and mucosal IgA responses when delivered with lactic acid bacteria [231]. This finding suggests that certain ARV components can be exploited to develop more effective vaccines and immunotherapies to protect poultry from ARV infections.

### 8.4. Immunoevasion Mechanism of ARV

It has been shown in several reports that dsRNA is a good inducer of the antiviral type I IFN system. Both positive- and negative-strand RNA viruses generate dsRNA intermediates during replication. While this is a natural byproduct for positive-strand viruses, it presents a unique challenge for dsRNA viruses like ARV, as dsRNA is a potent trigger of the host’s antiviral response. To avoid triggering an antiviral response, these viruses must prevent the cellular machinery from recognizing their dsRNA genomes. ARVs, like their mammalian counterparts, appear to achieve this by encoding the protein σA, which binds dsRNA [102], shielding the viral dsRNA from detection by cellular sensors like the PKR, which would otherwise trigger an antiviral response.

## 9. Disease Susceptibility and Transmission

The susceptibility and transmission dynamics of avian reovirus are influenced by several factors. Young chickens exhibit heightened susceptibility to infection and clinical signs compared to older birds. While older birds can become infected, they are generally less prone to developing clinical disease [177,178,232].

ARV can be transmitted both vertically, via the egg [179,180,181], and horizontally, primarily through the fecal–oral route [182], with fecal contamination of broken skin potentially serving as an additional route of ARV entry to leg tendons and joints [183]. ARV can persist in infected chicken legs for extended periods, up to 285 days [233]. Susceptibility to disease varies based on breed, conformation, and age, with chicks under two weeks of age being most susceptible [178,182]. While heavy meat-type chickens are frequently associated with reovirus-induced arthritis, light egg layers can also be infected and exhibit clinical signs [184]. Furthermore, broilers have been shown to be more susceptible to reovirus arthritis than white leghorn chickens [185].

## 10. Pathogenesis

ARV exhibits a broad tissue tropism following experimental infection in specific pathogen-free (SPF) chickens. Oral, intranasal, or intratracheal inoculation results in viremia and subsequent viral dissemination to various organs, including the respiratory, enteric, and reproductive tracts, as well as the hock joints and tendons [180]. Viral replication occurs primarily in the intestines and bursa of Fabricius, serving as a portal of entry for systemic spread [234]. The hock joint is another significant site of ARV replication [28,57,185]. In severe cases, ARV can infect the liver, leading to hepatitis and mortality [235]. The S1 gene segment, encoding the σC protein, plays a crucial role in determining ARV tissue tropism [236].

The incubation period of ARV infection varies depending on factors such as the age and breed of the infected bird, as well as the virus strain [1,137]. Experimental studies have shown incubation periods ranging from 1 to 13 days, with shorter incubation times observed for footpad inoculation and longer periods for intravenous, intramuscular, intratracheal, or contact infection [237]. Viral shedding typically peaks at 1–2 weeks post-infection and declines thereafter [238,239]. Oral shedding can persist for up to two weeks [240].

Field reports and experimental studies have indicated a diverse range of clinical manifestations associated with ARV infection in chickens. In broiler chickens, particularly those aged 3–4 weeks or 6–7 weeks, infection can lead to lameness, swollen joints, and retarded growth in birds (6–8%), as well as increased mortality rates along with seroconversion for reovirus antibodies in about 90% of cases [175,241,242]. Older birds, especially male chickens over 12 weeks of age, may experience lameness and tendon rupture [242,243]. Additionally, reovirus has been implicated in runting–stunting syndrome and malabsorption, although the specific mechanisms remain unclear [244,245].

**Figure 9:**
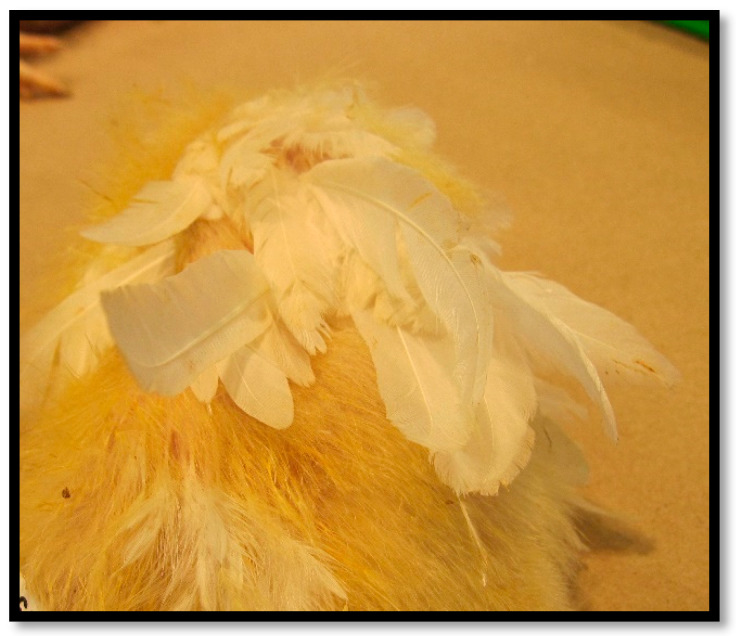
Abnormal development of primary feathers due to avian reovirus infection referred as helicopter wing feathers.

Some strains of avian reovirus (e.g., 2408 and 1733) have been shown to cause developmental abnormalities, such as stunting and feathering issues (Figure 9), when inoculated into young chicks (1 and 7 days old). Furthermore, neurological signs have been observed in specific experimental settings [246].

Histopathological examination of affected birds typically reveals intertarsal joint and tendon swelling as primary lesions. Synovial membranes may exhibit petechiae, and the joint articular surface can develop erosions. Joint cavities often contain increased volumes of non-turbid, straw-colored, or blood-tinted fluid. In chronic cases, fibrous adhesions can form between tendons and their sheaths, impairing normal joint function [247,248,249]. Experimental studies have demonstrated that reovirus infection can significantly weaken leg tendons, increasing their susceptibility to rupture, especially in older and heavier birds [175,247,248,249,250,251]. Moreover, experimental inoculation of 1-day-old chicks with reovirus can lead to systemic infection, affecting multiple organs, including the liver, spleen, kidney, bursa of Fabricius, heart, and tendons [252].

Viral tenosynovitis/arthritis in poultry was first documented in 1968, when Olson and Solomon [253] identified a novel viral agent associated with joint swelling and tendon inflammation in *Mycoplasma synoviae*-negative chickens. Subsequently, in 1980, Levisohn et al. reported a reovirus-induced tenosynovitis/arthritis in 15-week-old turkeys, characterized by swollen hock joints and histopathological evidence of synovial hyperplasia and inflammatory cell infiltration [44]. Page et al. (1982) further investigated the role of viral agents in turkey lameness, isolating viruses from affected birds that induced tenosynovitis when inoculated into young poults [45].

While the precise mechanisms underlying the development of viral tenosynovitis/arthritis in turkeys remain to be fully elucidated, it is postulated that the onset of clinical signs may be linked to a specific weight threshold, as suggested for viral arthritis in chickens [14]. However, Al Afaleq and Jones (1989) reported that reovirus strains isolated from turkeys and chickens with tenosynovitis/arthritis did not consistently induce the disease when inoculated into young poults, indicating a potential role of additional factors or specific viral strains in disease pathogenesis [46].

In recent decades, while TERV has emerged as a significant pathogen associated with various clinical manifestations, including diarrhea, poult enteritis, and light turkey syndrome [39,41], there have been no recent reports linking TERV to lameness or tenosynovitis/arthritis in turkeys.

### 10.1. Avian Reovirus Proteins and Host Pathogenesis

RNA viruses can be sensed at the plasma membrane or within endosomes via Toll-like receptors (TLRs). Alternatively, RNA viruses can be sensed in the cytoplasm by RIG-I-like receptors (RLRs) [254]. Engagement of TLRs or RLRs causes transcription factors’ activation, inducing the expression of antiviral effector proteins [254]. These proteins can either antagonize the viral replication cycle in the initially infected cell or can signal to neighboring cells via the production of cytokines, including interferons (IFNs), to prevent the establishment of infection [255]. In mammals, the two primary transcription factors that drive the innate immune response are NF-kB and interferon regulatory factor 3 (IRF3, absent in birds) [254]. These two transcription factors, acting either alone or in combination, control the expression of hundreds of target genes. Because these two transcription factors regulate such a wide variety of antiviral factors, they are the frequent targets of viral antagonism [256]. The mammalian reovirus μ1 protein (equivalent to µB in ARV) mediates membrane penetration and was found to regulate the activation of signaling cascades that culminate in cell death. This indicated that a diminished IRF-3, NF-kB, and apoptosis activation capacity was associated with reduced viral membrane penetration efficiency of host cells [257].

Conversely, research has identified the host protein IFN-γ-inducible protein-16 (IFI16) as an interacting partner of ARV P17 [258]. IFI16 belongs to the pyrin and HIN domain (PYHIN) containing protein family, which comprises crucial regulators of the innate immune response that detect microbial DNAs and dsRNA for inducing IFNs and/or activating inflammasomes [259,260]. Therefore, P17, playing a crucial role in viral replication and regulating cellular signaling pathways through its interaction with cellular proteins, could be related to viral arthritis or tenosynovitis and immunosuppression in chickens by recruiting inflammasomes to the infection site. Moreover, P17 was reported to induce retarded cell growth through activation of a p53-dependent pathway [115].

Another ARV protein found to play a role in pathogenesis was the FAST protein (P10). Previous studies [110,112] noted the similarity between the FAST proteins and a diverse group of viral membrane-interactive proteins, collectively coined viroporins [261]. Similar to the situation with viroporins, previous studies implicated the FAST proteins in reovirus egress. When ARV P10 trafficking to the plasma membrane is prevented, syncytium formation is inhibited, concurrent with delayed cell lysis and virus release [141]. The proposed membrane-lytic properties of P10 provide a possible explanation for the correlation between the extent of ARV-induced syncytium formation and viral pathogenesis [186], suggesting that the FAST proteins may function to promote cell lysis and virus release, thereby contributing to the natural pathogenicity of the fusogenic reoviruses [2,262,263]. Targeting these viral proteins could elucidate key molecular determinants of viral pathogenesis, facilitating the development of attenuated vaccine candidates with improved safety and efficacy.

### 10.2. Host Response to Avian Reovirus Infection and Clinical Outcomes

Viral pathogenesis encompasses a broad spectrum of diseases, including hepatitis, myocarditis, gastroenteritis, and immunosuppression, often leading to significant mortality rates. These pathologies may be linked to viral factors such as cell penetration, fusion, or the induction of apoptosis. Fusogenicity, mediated by the P10 protein in ARVs [186] and by a specific region in the glycoprotein B (C-terminal) of herpes simplex virus type 1, has been linked to increased pathogenicity in vivo through the formation of extensive syncytia [264,265]. Apoptosis has been implicated in ARV pathogenesis [142]. Additionally, apoptosis has been linked to various disease manifestations caused by other viruses [hepatitis C virus (HCV), severe acute respiratory syndrome coronavirus 2 (SARS-CoV2), Swine acute diarrhea syndrome coronavirus (SADS-CoV), mammalian reovirus (MRV) and transmissible gastroenteritis coronavirus (TGEV)], resulting in hepatitis (mediated by TNF, Fas, Bcl2-interacting killer (BIK), Caspases-3, 8, 9, and p53) [266], myocarditis (via TRIM29 or calpain induction) [267,268], immunosuppression (through IFN-λ suppression) [269], encephalitis (mediated by MRV μ1 activating NF-κB p50 subunit) [257], and gastroenteritis [270].

A direct link between ARV and tenosynovitis has not been conclusively demonstrated. The elevated expression of a gene, *WNT9a* (also known as *Wnt14*), was observed, which might play a key role in the development of the disease. While continued expression of *Wnt14* in mature joints may be beneficial for maintaining joint integrity, it has also been implicated in the development of rheumatoid arthritis in humans [271]. The up-regulation of *Wnt14*, combined with the induction of apoptosis [272], may be responsible for ARV-induced joint damage and more severe tendon rupture [47]. Another study linked the apoptosis of infected tendon cells to the infiltration of IFN-γ-expressing CD8+ T lymphocytes [273]. Furthermore, the severity of the disease, including gross and microscopic lesions, correlated with the ability of ARV variants to induce IFN-γ-producing CD8+ T lymphocyte recruitment to infected tendon tissues [273]. Identifying the viral factors that trigger apoptosis and attract these cytotoxic T cells to the gastrocnemius tendon could unveil novel therapeutic targets to ameliorate ARV-related disease symptoms.

### 10.3. Avian Reovirus-Induced Immune Suppression

Studies have yielded conflicting results regarding the effect of ARV infection on the immune system. Sharma and Fredericksen (1987) reported that pathogenic ARV strains, but not non-pathogenic ones, were associated with the depletion of lymphoid cells in the bursa and thymus and a reduced antibody response to an inactivated Newcastle disease virus (NDV) vaccine [274]. However, Montgomery et al. (1986) observed a decrease in bursa weight and some lymphocyte depletion but no significant impact on antibody responses to the NDV and Brucella abortus antigen [275]. Studies have shown that reovirus infection can suppress the response of peripheral blood monocytes and splenocytes to mitogens at 7 days post-infection (dpi) but not at later time points [227,275,276,277]. Pertile et al. further investigated and found that removing certain immune cells that adhered to plastic and produced nitric oxide (NO) partially restored the ability of T lymphocytes to respond to mitogens. This suggests that these plastic-adherent cells, possibly macrophages, might be acting as suppressor macrophages, dampening the overall immune response. Research in mammals suggests a different function of macrophage activation control. For instance, IL-13 appears to trigger the production of NO by macrophages [278]. On the other hand, IL-10, an acting suppressor of cytokine signaling-3 (SOCS3), seems to dampen the inflammatory response of macrophages [279]. These findings highlight the complex interplay between various factors in regulating macrophage activity. Additional research on the interactions of ARV proteins with cytokine pathways will be important to fully understand reovirus-induced immunosuppression in chickens.

## 11. Clinical Signs—Gross Lesions, Histopathology

Birds severely affected by reovirus infection exhibit pronounced swelling of the hock joints and enlargement of the gastrocnemius or digital flexor tendons [243]. In severe cases, birds may experience tendon rupture, particularly in heavier individuals, leading to immobilization and recumbency near water or feed sources. Bilateral tendon rupture results in a characteristic uneven gait due to the bird’s inability to mobilize the metatarsus, often accompanied by ruptured blood vessels.

**Figure 10:**
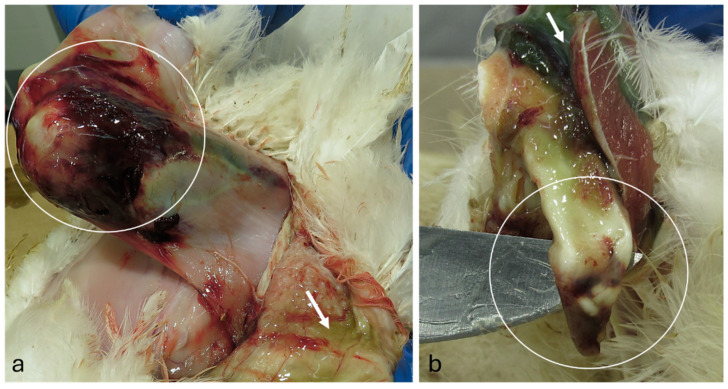
Hemorrhage and tendon rupture in the pelvic limb of broiler breeder chickens. (**a**) Severe hemorrhage surrounding the femorotibiotarsal joint (circled), with subcuticular edema (arrow). (**b**) Rupture of the flexor tendons at the level of the intertarsal joint (circled), with hemorrhage and edema extending into the surrounding muscle (arrow), which has been removed for visualization of tendon pathology.

The lesion is recognized by palpation just above the hock and can be easily demonstrated upon removal of feathers. The gastrocnemius tendon rupture is mostly characterized by a greenish skin discoloration owing to blood extravasation. Skin removal at necropsy will uncover the tendon-ruptured terminus (Figure 10) [185].

Grossly, naturally infected chickens display swelling of the gastrocnemius, digital flexor, and metatarsal extensor tendons. Enlargement of the shank below the hock may suggest digital flexor tendon swelling; however, necropsy typically reveals that the swelling is due to sigmoid folding and rupture of the flexor tendons at the hock level, accompanied by gelatinous fluid. Footpad and hock joint swelling is less common. The hock joint often contains a small amount of straw-colored or blood-tinged exudate, although in rare cases, a significant amount of purulent exudate may be present, resembling that seen in mycoplasma synovitis. Early infection is marked by edema of the tarsal and metatarsal tendon sheaths, along with frequent petechial hemorrhages in the synovial membranes above the hock joint.

Inflammation of the tendon areas progresses to a chronic phase characterized by tendon sheath hardening and fusion. Minor pitted erosions develop in the distal tibiotarsal articular cartilage, which may increase, merge, and extend into the underlying bone. An overgrowth of fibrocartilaginous pannus develops on the articular surface, frequently involving the condyles and epicondyles [30]. The proximal metatarsal diaphysis of the affected limb can be inflamed as well.

**Figure 11:**
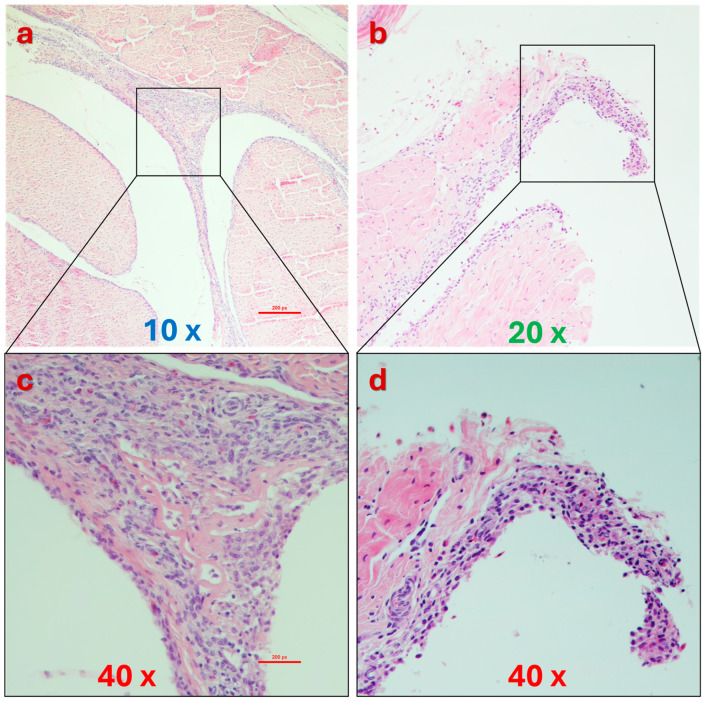
Histopathology in tendons of ARV-infected broilers. Tendon tissues showed mild to moderate multifocal thickening of the synovium with mononuclear inflammatory cell infiltrates and synovial hyperplasia (**a**,**b**). (**c**,**d**) are higher magnifications of the boxed areas in (**a**,**b**), respectively.

Histopathologically, acute-phase reovirus infections (1–2 weeks post-footpad inoculation) manifest as edematous gastrocnemius tendon sheaths, synoviocyte hyperplasia (Figure 11), and a lymphocytic and macrophagic infiltrate within the subsynovium [175,177]. As the infection progresses to the chronic phase, the hallmark becomes fibroplasia and the accumulation of fibrous connective tissue in the subsynovium, accompanied by villous-like synovial processes. Periostitis and increased osteoclast activity may also be evident during this stage.

Oral reovirus inoculation in chickens can induce gastrocnemius tendon and sheath fibrosis, leading to adhesion and joint immobilization by 7–8 weeks post-infection [52]. Reovirus infections have been associated with hock joint articular cartilage erosion and subchondral bony exostosis, further contributing to joint immobilization [30]. While the histologic presentation of reovirus tenosynovitis may resemble that of *Staphylococcus aureus* or *Mycoplasma synoviae* infections [14], a key distinguishing feature is the presence of lymphocytic inflammation in reovirus infections, whereas *Mycoplasma* and *Staphylococcus* infections typically exhibit caseous inflammation [251].

**Figure 12:**
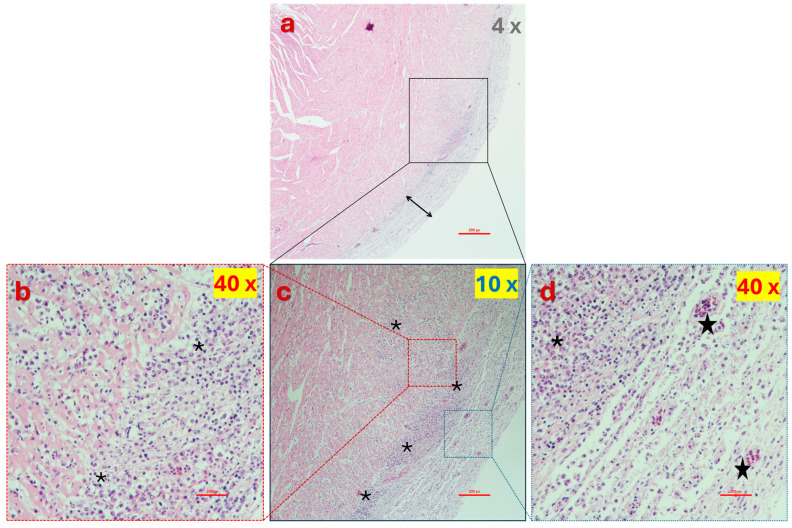
Histopathology of the heart of ARV-infected broilers. Heart tissue demonstrated moderate diffuse thickening of the pericardium ((**a**), pericardium delineated by a double-headed arrow) characterized by accumulations of fibrin, edema, vascular congestion (starred), and lymphoplasmacytic infiltration (asterisks) with multifocal extension into the myocardium (**b**) and epicardium (**d**). (**c**) is a higher magnification of the squared area of subfigure (**a**).

Reovirus infections can extend beyond the musculoskeletal system to affect visceral organs. Myocarditis and pericarditis, characterized by heterophilic and lymphocytic infiltration of the myocardium and lymphocytic aggregates in the epicardium, have been observed in infected chickens (Figure 12) and are considered nearly pathognomonic for reovirus arthritis [30,253,280]. Additionally, hepatic necrosis has been reported in chickens infected with reovirus at one day of age [281]. Ultrastructural studies have revealed fibroblastic lesions within the gastrocnemius tendon and sheath of orally infected broilers, including cytoplasmic vacuolization, ribosomal loss from the endoplasmic reticulum, and mitochondrial disruption, between 1 and 5 weeks post-infection [251].

## 12. Diagnosis

### 12.1. Localization of Viral Antigens and Nucleic Acids in Tissue

To identify the presence of reovirus in infected tissues, immunohistochemical staining techniques can be employed on formalin-fixed paraffin-embedded samples to detect viral proteins [282]. In situ hybridization (ISH) is another valuable tool that allows for the visualization of viral nucleic acids within tissue sections [283]. Probes designed to target highly conserved regions of the viral genome can be utilized to detect a broad range of reovirus strains. For rapid diagnosis during the early stages of infection, fluorescent antibody staining can be performed on cryostat sections of fresh-frozen tissues, such as tendon sheaths, to detect viral antigens [284].

### 12.2. Avian Reovirus Isolation

ARV, while abundant, is often non-pathogenic. Isolating the virus solely from the intestinal tract may not be sufficient to definitively link it to joint lesions. Conversely, isolation from hock joint tissues is more indicative of a causal relationship. However, viral isolation from advanced-stage joint lesions may prove challenging. Optimal tissues for isolation include the hypotarsal sesamoid bone with its associated tendons, synovial membrane, and articular cartilage. Samples can be stored short-term at 4 °C or long-term at −20 °C or below [193]. For joint-specific isolation, tissue samples are generally more informative than swabs. The hypotarsal sesamoid bone with its tendons, synovial membrane, and articular cartilage are the preferred tissues. Sampling both infected and apparently healthy birds is crucial, as clinical signs may be subtle, and viral detection can precede lesion development.

ARV propagates efficiently in embryonated chicken eggs via yolk sac or chorioallantoic membrane (CAM) inoculation [33,186,285], resulting in mortality within 3–5 or 7–8 days post-inoculation, respectively [193]. Cell culture propagation is also feasible in various cell lines, including Vero (African green monkey kidney), LMH (chicken liver), BHK-21 (baby hamster kidney), CRFK (Crandell feline kidney), RK (rabbit kidney), GBK (Georgia bovine kidney), QT35 (quail muscle), PK-15 (porcine kidney), chicken lymphoblastoid cells, primary chicken kidney cells, and primary chicken embryo liver cells [17,193,286]. While chicken embryo fibroblasts can be used, adaptation may be necessary. Reovirus infection in chicken-origin cell cultures is characterized by syncytia formation within 24–48 h, followed by monolayer degeneration and the formation of giant cells. Intracytoplasmic inclusions, either eosinophilic or basophilic, are also observed in infected cells. Post-isolation identification can be achieved through electron microscopy, immunofluorescence, RT-PCR, or sequencing.

### 12.3. Pathogenicity Determination of Variant Avian Reovirus Strains

Despite extensive molecular investigations, reliable markers for ARV pathogenicity remain elusive. A traditional approach to confirm the arthrotropic potential of an ARV isolate involves footpad inoculation of day-old susceptible chicks. Pathogenic ARVs induce a marked inflammatory response in the footpad within 72 h post-inoculation [287]. Alternatively, oral infection, while more natural, requires a longer observation period.

ARV variant strains exhibit a propensity to replicate and induce gross and microscopic lesions within the pericardium, tendons, and tendon sheaths [288,289], with varying degrees of severity influenced by the specific ARV strain. Additionally, ARV variant infections have been linked to varying levels of bursal and thymic lymphoid depletion, suggesting a potential role in immunosuppression in infected chickens [288].

### 12.4. Serological Assays

Serological methods, particularly ELISA, are widely used to assess the immune status of poultry flocks against ARV infections [290]. While these assays are efficient for detecting ARV antibodies, they lack specificity for other reovirus serotypes like TERV and TARV. To address this limitation, ELISAs have been developed using various antigens, including whole virus, recombinant σC, σB, and σA proteins [290,291]. Although a strong correlation exists between ELISA-determined antibody levels and virus-neutralizing antibodies [238], the widespread prevalence of multiple ARV serotypes in commercial flocks often complicates the interpretation of serological profiles.

To overcome these challenges, DIVA strategies have been employed. ELISAs using variable regions of σC or nonstructural proteins as antigens have been developed to differentiate between infected and vaccinated birds [292,293]. However, the gold standard for serotyping ARV remains the virus neutralization test [129].

ARVs exhibit significant antigenic diversity. In the 1960s, Japanese ARV strains were classified into five serotypes using plaque reduction tests [294,295]. Subsequent studies in the 1980s involving strains from the United States, Germany, Japan, and Great Britain further confirmed serotypic differences and highlighted the variability and subjectivity of neutralization assays [296]. Australian researchers in the 1980s identified three distinct subtypes within a single serotype, suggesting a complex antigenic relationship [292]. Moreover, investigations in the 1980s revealed the emergence of antigenic variants that did not cross-react with the S1133 vaccine strain, emphasizing the potential for vaccine failure [130].

### 12.5. Molecular Approaches

Molecular methods of particular interest, RT-PCR, have become the gold standard for ARV detection due to their rapid, specific, and sensitive nature [297]. These techniques have been employed for various applications, including vaccine screening [298], simultaneous detection of multiple avian viruses [299], and universal detection of all ARVs or reference strains across different avian species [300]. Recent advancement includes the development of one-step RT-PCR for turkey reovirus detection [301].

Genetic characterization of ARV isolates typically involves sequencing the σC gene, followed by bioinformatics analysis (strain variation). For a more comprehensive understanding of emerging ARV strains, next-generation sequencing (NGS) can be utilized for full genomic characterization [17,286]. While the current molecular classification system based on the σC gene provides valuable epidemiological insights, it falls short of providing comprehensive information on the antigenicity and pathogenicity of ARV variants, hindering the development of effective prevention strategies. Traditional antigenicity and pathogenicity studies are impractical and do not meet the need for a robust ARV typing method.

To address these limitations, a novel approach is required to correlate gene sequences with viral antigenicity and pathogenicity. This strategy has been successfully applied to other Reoviridae family members, such as rotaviruses, where the VP4, VP6, and VP7 genes have been used for genotyping and providing antigenic and pathogenic information [302]. Rotavirus classification is based on the VP6 protein, which determines antigenic group and subgroup, and the VP4 and VP7 proteins, which are involved in serotype specificity. A similar classification system could be beneficial for ARVs. By analyzing the sequences of relevant genes, it may be possible to predict the antigenicity of both outer and inner capsid proteins, providing a more comprehensive understanding of ARV diversity. In avian reoviruses, the L3, M2, and S1 genes are promising candidates for a comprehensive classification system [17,288]. These genes encode proteins that are located at different levels of the virion and exhibit significant genetic variability, suggesting their potential role as antigenic determinants [193,288].

## 13. Prevention and Control—Biosecurity and Vaccine

ARV is ubiquitous in commercial poultry production, with prevalence rates approaching 100% [303]. Despite the challenges associated with maintaining ARV-free flocks in modern, intensively housed environments, implementing rigorous biosecurity measures can significantly reduce infection prevalence. These measures include minimizing exposure to contaminated feed and water sources, which facilitate the fecal–oral transmission of ARV, as well as avoiding multi-age farms that can serve as reservoirs for viral circulation between younger and older birds. While ARV is relatively stable, multi-component disinfectants have proven effective in inactivating the virus [304]. Thorough cleaning and disinfection of affected barns is also crucial for effective biosecurity.

The control of viral arthritis in poultry is primarily achieved through the vaccination of breeder flocks to induce high levels of neutralizing anti-reovirus antibodies. These antibodies serve a dual purpose: preventing maternal infection and vertical transmission to offspring via the yolk sac and providing passive immunity to the progeny [217,305,306]. The critical period for pathogenic reovirus infection is the first few days of life when maternal antibodies wane and become non-protective at 10–15 days old [189,294]. The half-lifetime of maternal antibodies in chicks is around 5 days [294]. Appropriate vaccination of broiler breeders can significantly enhance both the initial levels and duration of maternal antibody protection in chicks [307].

Live attenuated and inactivated vaccines are available for reovirus control. Commercially licensed live reovirus vaccines in the United States primarily utilize the S1133 strain, with the exception of 2177^®^, both belonging to reovirus genotype 1. The S1133 strain required extensive attenuation through 235 serial passages in embryonated chicken eggs, followed by 100 passages in chicken embryo fibroblast cultures to achieve a safety profile suitable for young chickens [308]. Inactivated vaccines, on the other hand, typically contain combinations of S1133, 1733, 2408, and/or Miss B strains [309]. A notable exception is Avian Reovirus Vaccine™, which incorporates antigenic variant reovirus serotypes 1/4455, 2/4455, and 3. Due to the antigenic disparity between the commercially licensed vaccines and contemporary field strains, these vaccines offer limited protection against current avian reovirus challenges (excluding the recent addition of Avian Reovirus Vaccine™). This lack of homologous vaccine options has led to the widespread use of autogenous inactivated reovirus vaccines within the U.S. broiler industry derived from circulating field strains due to the emergence of antigenically distinct variants [16,132]. These autogenous vaccines require regular updates to maintain efficacy against evolving viral genotypes [130]. Combination vaccines incorporating multiple genotypic representatives can provide broader protection [287].

While there is no standardized vaccination protocol, a common approach involves a combination of live attenuated and inactivated vaccines [310]. Live vaccines should be administered before egg production to avoid trans-ovarian transmission of the vaccine virus [295]. Broiler breeders are typically vaccinated with 1–3 live attenuated vaccines up to 12 weeks of age, followed by 1–3 inactivated vaccines [193]. These vaccines are administered intramuscularly or subcutaneously to protect against tenosynovitis and malabsorption.

The efficacy of reovirus vaccines is commonly assessed through a challenge model involving footpad inoculation of day-old chicks with a virulent autogenous virus. While this method can be informative, interpretation of results can sometimes be challenging. Novel vaccination approaches, such as in ovo vaccination [311] and subunit vaccines based on viral proteins like σC and σB [312,313,314], have shown promise but are not yet widely implemented. For turkeys, polyvalent autogenous vaccines have been effective in reducing the prevalence of turkey viral arthritis. However, commercial vaccines specifically for turkeys are not currently available.

Recombinant poultry vaccines based on viral vectors, fowlpox virus, and turkey herpesvirus (HVT) [315] have been developed and commercialized. The recombinant vector vaccines offer significant safety advantages over live attenuated vaccines. They are genetically stable and cannot revert to virulence. In ovo vaccination remains an attractive immunization approach for the poultry industry. It has many advantages, such as early immune response and low labor costs, and it can quickly and uniformly mass vaccinate large chicken populations [316]. Among all the vector vaccines, HVT vector vaccines are particularly well-established, allowing for subcutaneous administration on the day of hatch or in ovo at embryonic day 18 [317,318,319]. These vaccines exhibit limited horizontal transmission within the flock. In addition, HVT can offer dual protection against Marek’s disease (MD) and the expressed transgene(s) of the target pathogen. The current MD vaccine, composed of HVT and MDV serotype 2 vaccines (e.g., SB-1, 301B/1), offers synergistic protection against very virulent pathotypes of MDV [320,321].

## 14. Current Challenges, Gap in Knowledge and Conclusions

While significant progress has been made in ARV research, substantial knowledge gaps remain in our understanding of ARV pathogenesis. These include (a) the pathological effects of circulating strains; (b) the molecular determinants of virulence that mandate a full understanding of the specific functions of ARV proteins and their contribution to disease progression; (c) the precise molecular mechanisms underlying specific conditions, such as arthritis, tenosynovitis, and hepatitis; (d) the complex interplay between the virus and the host immune response in the development of various ARV disease manifestations; (e) the mechanisms by which ARV evades the host immune response; (f) the potential for host tropism and cross-species transmission; (g) the factors influencing the efficacy of different vaccine types against diverse ARV strains, the efficacy of existing vaccines, and the need for vaccine updates to incorporate newly identified strains; and (h) the specific immune responses required for long-lasting protection against ARV infection. Many of these unanswered questions have been hindered by the lack of a reverse genetics system for ARV.

In conclusion, ARV poses a significant economic burden on the global poultry industry due to its widespread prevalence and diverse clinical manifestations. While significant advancements have been made in understanding the molecular virology, pathogenesis, and epidemiology of ARV, challenges persist in the development of effective control strategies. Continued research efforts are crucial to identify novel vaccine candidates, develop advanced diagnostic tools, and implement robust biosecurity measures to mitigate the impact of ARV infections on poultry health and production. A comprehensive approach involving a combination of these strategies is necessary to address the complex nature of ARV infections and safeguard the poultry industry.

## Figures and Tables

**Table 1:** Avian reovirus genotypes and associated clinical profile.

Genotype	Identity %	Subgroups	Highest Prevalence	Clinical Signs	References
GCI	>80%	3–4	2016–2017	Tenosynovitis/arthritis, runting–stunting syndrome, malabsorption syndrome, respiratory disease,	[106,132,198,200,201,206,208]
GCII	≥70%	4	2020–2021	mild tenosynovitis and variable uniformity issues and malabsorption	[106,198,200,201,208]
GCIII	>80%	2–3	2021	Tenosynovitis/viral arthritis, variable uniformity, hydropericardium, and maldigestion	[53,106,198,200,201,208]
GC IV	≥70%	4	2017	Severe crippling of birds, tenosynovitis/arthritis, runting–stunting syndrome, Malabsorption syndrome	[106,198,201,208]
GC V	83%	3	2012–2014	Significant tenosynovitis/lameness and swelling of the digital flexor tendons, runting–stunting syndrome, hepatitis, myocarditis, and central nervous system disease	[48,106,198,200,201,208]
GC VI	>70%	2	2020	Tenosynovitis/viral arthritis	[16,17,48,190,198,208,213,214]
GC VII	>70%	1	2019	-	[198]

## Data Availability

All the data are available within the manuscript.
